# Polymeric Theranostics with Tetraphenylporphyrin for Effective Low-Dose Photodynamic Cancer Therapy

**DOI:** 10.3390/pharmaceutics18050531

**Published:** 2026-04-27

**Authors:** Alžběta Turnovská, Shanghui Gao, Marina Rodrigues Tavares, Jan Hynek, Kamil Lang, Jun Fang, Tomáš Etrych

**Affiliations:** 1 Institute of Macromolecular Chemistry, Czech Academy of Sciences, 162 00 Prague, Czech Republic; turnovska@imc.cas.cz (A.T.); tavares@imc.cas.cz (M.R.T.); 2 Laboratory of Microbiology and Oncology, Faculty of Pharmaceutical Sciences, Sojo University, Kumamoto 860-0082, Japan; gaoshanghui94@gmail.com; 3 Institute of Inorganic Chemistry, Czech Academy of Sciences, 250 68 Husinec-Řež, Czech Republic; hynek@iic.cas.cz (J.H.); lang@iic.cas.cz (K.L.); 4 Department of Toxicology, School of Public Health, Anhui Medical University, Hefei 230032, China

**Keywords:** photodynamic therapy, polymer drug delivery, sarcoma, polymer–photosensitizer conjugate, tumor-targeted nanomedicine

## Abstract

**Background/Objectives**: Photodynamic therapy (PDT) relies on light activation of photosensitizers to generate reactive oxygen species for tumor ablation; however, limited tumor selectivity and systemic toxicity of free photosensitizers remain challenges. This study aimed to develop polymer-based nanotheranostics carrying tetraphenylporphyrin (TPPc) derivatives and to evaluate how linker structure impacts their performance. **Methods**: TPPc derivatives were covalently conjugated to *N*-(2-hydroxypropyl)methacrylamide (HPMA)-based polymers via either pH-sensitive hydrazone linkages (using aliphatic 5-hydroxy-2-pentanone or aromatic 1-(4-hydroxymethyl)phenyl)ethanone spacer) or stable amide bonds, forming amphiphilic polymer conjugates. The conjugates were characterized based on their physicochemical and in vitro and in vivo biological behavior. **Results**: Polymer conjugation reduced dark toxicity while preserving photodynamic activity. Linker structure influenced intracellular behavior and singlet oxygen production, with hydrazone systems showing faster activation-related responses under acidic conditions in vitro. All conjugates accumulated in tumors and induced significant tumor growth inhibition after irradiation at low doses (2.5 mg kg^−1^ TPPc equivalent), while the amide-linked conjugate showed the strongest overall in vivo therapeutic effect, likely due to more favorable biodistribution and sustained delivery. **Conclusions**: The developed HPMA-based polymer–TPPc conjugates improve the therapeutic profile of photosensitizers by reducing toxicity and enabling effective PDT. These findings highlight the importance of linker design in balancing photosensitizer activation, circulation stability, and biodistribution, which together determine the overall therapeutic outcome.

## 1. Introduction

Photodynamic therapy (PDT) is an established treatment modality that combines a photosensitizer (PS), light, and oxygen to induce localized oxidative damage in tumor tissues. Despite its clinical potential, the broader application of PDT remains limited by the physicochemical properties of many PSs, particularly their poor water solubility, aggregation in biological media, and suboptimal pharmacokinetics. These factors reduce effective singlet oxygen generation and hinder efficient delivery to tumor tissue [1].

Porphyrins, such as derivatives of 5,10,15,20-tetraphenylporphyrin (TPP), are widely used as PSs in PDT, due to their photophysical properties, structural stability [2,3] and relatively easy synthesis [4,5]. Importantly, while some previously investigated PSs, such as zinc protoporphyrin IX or pyropheophorbide-a (PyFa), exhibit lower photocytotoxicity compared to clinically used agents like temoporfin (mTHPC), monosubstituted tetraphenylporphyrins have demonstrated comparable activity, highlighting the significant therapeutic potential of TPP-based systems in PDT [6,7,8]. These properties, together with their synthetic versatility, make TPP-based systems suitable platforms for investigating structure–activity relationships, particularly in the context of polymer-based delivery systems.

Upon light irradiation, the PS interacts with surrounding substrates or molecular oxygen generating reactive oxygen species (ROS)—such as singlet oxygen O_2_(^1^Δ_g_)—responsible for oxidizing proteins, carbohydrates, DNA, lipids, and other biologically important molecules, causing oxidative stress and initiating biological cascades, subsequently leading to cell death [9,10,11,12]. The antitumor effect of PDT arises from a combination of direct tumor cell killing, damage to tumor vasculature, and activation of immune responses. Due to its localized activation by light, PDT enables controlled treatment of tumor tissue with limited damage to surrounding healthy structures [13,14,15]. Because these cytotoxic effects are mediated by short-lived reactive species, the therapeutic outcome of PDT critically depends on the effective delivery, availability, and activation of the photosensitizer at the target site, making its physicochemical properties and biodistribution critical factors for treatment efficacy. PDT is also limited by the hydrophobicity of many photosensitizers, their low solubility in aqueous environments, and their tendency to aggregate, which leads to self-quenching and reduced ROS generation [16,17].

Carrier platforms (with liposomes, lipid nanoparticles, oil dispersion, or polymeric nanoparticles having been reported [18]) have been widely explored to address these challenges by improving PS solubility and circulation behavior, often enhancing tumor accumulation via the enhanced permeability and retention (EPR) effect [19,20,21]. However, many reported systems rely on physical encapsulation or non-specific loading, which may lead to premature release, uncontrolled distribution, or reduced photodynamic efficiency due to aggregation. In contrast, covalent conjugation of PSs to polymer carriers offers greater control over stability and biodistribution, but introduces a new challenge: balancing sufficient stability during circulation with effective activation at the target site [22].

In this context, the design of the linker between the PS and the carrier represents a critical parameter. Linker chemistry can influence not only the release profile of the PS, but also its intracellular availability and photodynamic activity. In particular, pH-sensitive linkers such as hydrazones may enable controlled release in acidic tumor environments, whereas stable linkages such as amides can ensure prolonged retention of the PS within the carrier system. The relative contribution of these two design strategies to overall therapeutic efficacy, however, may depend on the balance between release, de-aggregation, biodistribution, and tumor accumulation.

**Figure 1:**
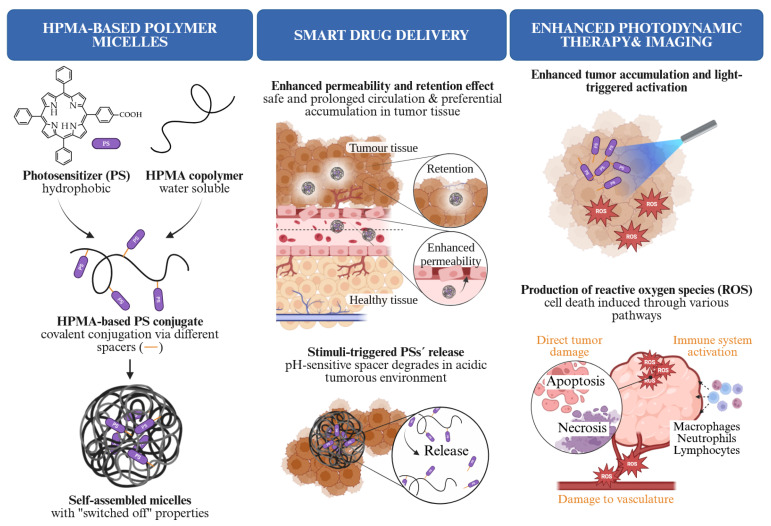
Graphical representation of the work concept. Created in BioRender.

In this work, a series of *N*-(2-hydroxypropyl)methacrylamide (HPMA)-based polymer–PS conjugates with systematically varied linker and spacer structures was biologically evaluated with the aim of studying the performance of developed nanomedicines during the PDT of solid tumors. Nanomedicines with TPP derivatives bearing different spacer moieties covalently attached to the polymer backbone via either pH-responsive hydrazone bonds (with aliphatic or aromatic character) or non-cleavable amide linkages were evaluated. The resulting amphiphilic conjugates formed self-assembled micellar nanosystems with high stability in aqueous environments. This design enables direct comparison between systems with distinct release behaviors, ranging from partial pH-triggered release under mildly acidic conditions to complete stability under physiological conditions. Through this approach, the influence of linker and spacer design on self-assembly, stability, release profile, and subsequent photodynamic activity was systematically evaluated. This work addresses how molecular design of the conjugate affects both PS activation and biodistribution, rather than focusing on a single determinant of efficacy. The results demonstrate that different conjugation strategies affect singlet oxygen generation and overall therapeutic outcome, with the prepared systems exhibiting significant antitumor efficacy even at low doses. These findings provide a basis for the rational design of polymer-based PDT nanomedicines. The overall concept of this work is graphically represented in Figure 1.

## 2. Materials and Methods

### 2.1. Chemicals

2,2′-azobisisobutyronitrile (AIBN), methacroyl chloride, 1-aminopropan-2-ol, 6-aminohexanoic acid, *N*-(3-dimethylaminopropyl)-*N*′-ethylcarbodiimide hydrochloride (EDC), carbon disulfide, ethanthiol, sodium hydride (60% dispersion in mineral oil), 5-hydroxy-2-pentanone, 2-thiazoline-2-thiol (TT), 4,6-trinitrobenzene-1-sulfonic acid (TNBSA), 4-(dimethylamino)pyridine (DMAP), *N*,*N*-diisopropylethylamine (DIPEA), 4,4′-azobis(4-cyanovaleric acid) (ACVA), 4-(2-carboxyethylsulfanylcarbothioylsulfanyl)-4-cyanopentanoic acid (carboxyethyl-TTc-ACVA), *tert*-butanol (*t*-BuOH), *N*,*N*-dimethylacetamide (DMA), dimethyl sulfoxide (DMSO), dichloromethane (DCM), 1,4-dioxane, Tween-20, and lithium bromide (LiBr) were obtained from Merck (Prague, Czech Republic). 1-(4-hydroxymethyl)phenyl)ethanone was obtained from abcr GmbH (Karlsruhe, Germany). *N*-(3-Boc-aminopropyl)methacrylamide (Ma-AP-NH-Boc) was obtained from Polysciences, Inc. (Warrington, PA, USA). Methanol (MeOH), acetic acid, chloroform (CHCl_3_), hydrochloric acid (HCl), N-hexane, and ethyl acetate were obtained from Lach: Ner (Neratovice, Czech Republic). Azoinitiator 2,2’-azobis(4-methoxy-2,4-dimethylvaleronitrile) (V-70) was obtained from Wako Pure Chemical Industries Ltd. (Osaka, Japan). Trifluoroacetic acid (TFA) was purchased from Iris Biotech GmbH (Marktredwitz, Germany). The spin-trapping agent, 2,2,6,6-tetramethyl-4-piperidone (TMP), was purchased from Tokyo Chemical Industry, Tokyo, Japan. Ethanol (EtOH), *N*,*N*-dimethylformamide (DMF), acetonitrile (ACN), as well as the solvent for the nuclear magnetic resonance (NMR) spectroscopy, DMSO-*d*_6_ (99.80 atom% D), were obtained from VWR Chemicals (Leuven, Belgium). All solvents used were of analytical grade. Column chromatography was performed on silica gel (0.060–0.200 mm, 60 Å) purchased from Thermo Scientific (Brno, Czech Republic).

Acetate-TT was synthesized by means of carbodiimide chemistry from acetic acid by reaction with 2-thiazoline-2-thiol in the presence of EDC in DCM as described previously [23]. 5,10,15-triphenyl-20-(4-carboxyphenyl) porphyrin (TPPc) was synthesized using the statistic condensation method [24]. Detailed procedure describing the synthesis of initial chemicals can be found in the literature [23].

### 2.2. Synthesis of Monomers and CTA

HPMA was prepared as described in the literature [25]. *N*-(tert-butoxycarbonyl)-*N*′-(6-methacrylamidohexanoyl)hydrazine (Ma-Ahx-NHNH-Boc) was synthesized by two-step synthesis from Ma-Ahx-COOH, described elsewhere [26]. The trithiocarbonate chain transfer agent *S*-2-cyano-2-propyl-*S*′-ethyl trithiocarbonate (AIBN-TTc) was synthesized according to Ishitake et al. [27].

### 2.3. Synthesis of TPPc Derivatives (dTPPs)

**Figure 2:**
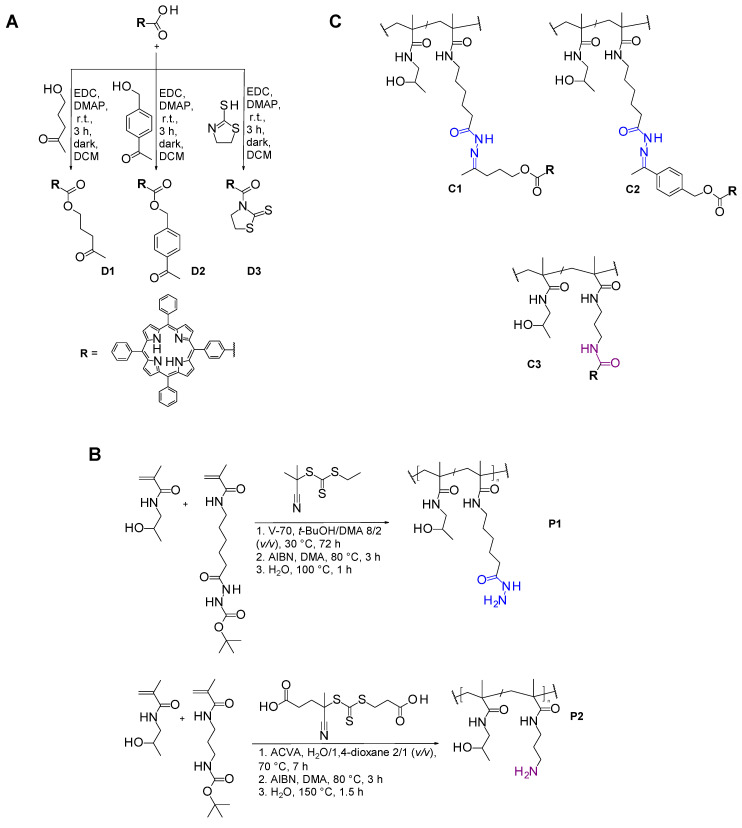
Scheme of the synthesis of dTPPs (**A**), polymer precursors for subsequent linkage of dTPPs (**B**) and structures of the final polymer conjugates (**C**).

Carbodiimide chemistry was used for the transformation of TPPc to its derivatives (dTPPs) for a subsequent formation of hydrazone or amide bonds with the polymer precursors **P1** and **P2**, respectively. TPPc, EDC, DMAP, and either 5-hydroxy-2-pentanone or 1-(4-hydroxymethyl)phenyl)ethenone were mixed for the preparation of derivatives **D1** with aliphatic spacer or **D2** with aromatic spacer, respectively. For the preparation of derivative **D3**, the carboxylic group of TPPc was transformed into a more reactive 2-thiazoline-2-thiol amide by dissolving TPPc, along with EDC, 2-thiazoline-2-thiol, and DMAP. The preparation of derivatives **D1**–**D3** was carried out in DCM under continuous stirring in the dark at room temperature. The synthesis was described in more detail previously [23]. The reactions were monitored using thin-layer chromatography (TLC) on TLC Silica gel 60 F_254_ (Merck, Czech Republic) with hexane/ethyl acetate 2/1 (*v*/*v*) as a mobile phase, and silica gel (60 Å) column chromatography was used to purify the products with a gradient of hexane/ethyl acetate from 7/1 to 1/1 (*v*/*v*). Compounds **D1**–**D3** were characterized by ^1^H NMR and matrix-assisted light desorption/ionization (MALDI) analysis [23]. The dried droplet method was used to prepare samples in DCM for MALDI measurements. The spectra were acquired with an UltrafleXtreme TOF mass spectrometer (Bruker Daltonics, Bremen, Germany) equipped with a 2000 Hz smartbeam-II laser (355 nm) in a positive ion reflection mode. The detailed reaction schemes are shown in Figure 2A.

### 2.4. Synthesis of Polymer Precursors

Radical reversible addition–fragmentation chain transfer (RAFT) polymerization was used for the synthesis of both polymer precursors: **P1** p(HPMA-*co*-Ma-Ahx-NHNH_2_) with hydrazide groups and **P2** p(HPMA-*co*-Ma-AP-NH_2_) with amine groups alongside the main chain.

For **P1**, HPMA and Ma-Ahx-NHNH-Boc monomers were dissolved in 80/20 (*v*/*v*) *t*-BuOH/DMA mixture with 95/5 molar ratio, in the presence of V-70 initiator and AIBN-TTc CTA and left to polymerize for 72 h at 30 °C. The monomers/CTA/initiator molar ratio was set at 225/1/0.5. Polymer precursor **P2** was prepared by dissolving HPMA and Ma-Ap-NH-Boc with ACVA initiator and carboxyethyl-TTc-ACVA CTA. The reaction was carried out in a distilled water/1,4-dioxane mixture (2/1) at 70 °C for 7 h. The ratio of monomers HPMA/Ma-Ap-NH-Boc was 96/4 and the monomers/CTA/initiator ratio was 225/1/0.5. CTA end-group removal was carried out by reaction of polymers with an excess of AIBN at 80 °C for 3 h [28]. Functional hydrazide (**P1**) or amine (**P2**) groups were thermally deprotected in Q-water [29]. Final polymer precursors **P1** and **P2** were obtained by lyophilization. Content of functional groups was determined using UV/VIS spectrophotometry. The characterization of both **P1** and **P2** polymer precursors is summarized in Table 1. The detailed synthetic scheme is depicted in Figure 2B. The detailed procedure of polymer precursor preparation and characterization are described elsewhere [23].

### 2.5. Synthesis of Polymer Conjugates

Polymer precursor **P1** with -NHNH_2_ groups was used for the attachment of **D1** and **D2**, yielding polymer conjugates with degradable hydrazone bonds, **C1** and **C2**, with an aliphatic and aromatic spacer, respectively. Polymer precursor **P1** with either **D1** or **D2** derivative were dissolved in MeOH/DMA 8/2 (*v*/*v*) in the presence of acetic acid and the reaction mixture was left overnight in the dark at room temperature under continuous stirring. Detailed synthesis was described previously [23].

Polymer precursor **P2** with -NH_2_ groups was used for the attachment of **D3**, in the presence of DIPEA, resulting in a conjugate with a stable amide bond. Due to residual potentially cytotoxic -NH_2_ groups, part of the stable conjugate was left as it was (**C3WA**, with residual amines) and the rest was reacted with an excess of acetate-TT for their effective protection. The resulting protected material is denoted as **C3** [23]. The cytotoxicity caused by residual amine groups (**C3WA** vs. **C3**) was evaluated (Figure S2). All further experiments were carried out with **C3** with protected amine groups.

The purification procedure, analogous for all conjugates, involved repeated precipitation into ethyl acetate/CHCl_3_ 5/2 (*v*/*v*) mixture followed by filtration, washing of the precipitate with ethyl acetate/diethyl ether 1/1 (*v*/*v*) mixture, and drying under vacuum. Purity of the conjugates was evaluated by TLC using hexane/ethyl acetate 2/1 (*v*/*v*) as a mobile phase and by size exclusion chromatography. The structure of the conjugates and the reaction schemes are presented in Figure 2C.

### 2.6. Dynamic Light Scattering (DLS)

Dynamic light scattering (Zetasizer Ultra, Malvern Panalytical, Worcestershire, UK) was used for determination of hydrodynamic diameters (*D*_H_) of polymer precursors **P1** and **P2** and polymer conjugates **C1**–**C3** at λ = 632.8 nm and θ = 173°, with the refractive index set as 1.59. The Zetasizer “ZS XPLORER” 4.0.0 software was used for data evaluation. A fluorescence filter was applied during the measurement of the conjugates. Hydrodynamic diameters were measured in Q-water (refractive index 1.33) with a concentration of 3.0 mg mL^−1^ for **P1** and **P2** and in phosphate buffer of pH 7.4 /EtOH 5% (*v*/*v*) (refractive index 1.22) with a concentration of 1.0 mg mL^−1^ for **C1**–**C3**. DLS was also used to evaluate the changes in *D*_H_ of conjugates upon release of dTPPs. The measurement was performed in PB of pH5.0/DMSO (5% (*v*/*v*)) with a concentration of 2.0 mg mL^−1^ using fluorescence filters.

### 2.7. Size Exclusion Chromatography (SEC)

Size exclusion chromatography was used for the determination of number-average molecular weight (*M*_n_), weight-average molecular weight (*M*_w_), and dispersity (*Đ*) of polymer precursors **P1** and **P2** as well as for the determination of the purity of **C1**–**C3** conjugates. An HPLC Shimadzu system equipped with a refractometric detector (Optilab, Wyatt, Dernbach, Germany), a photodiode array UV-VIS detector (SPD-M40, Shimadzu, Kyoto, Japan) and an 8-angle light scattering detector (DAWN, Wyatt, Germany) was used. The analysis was performed with DMF + LiBr (10 mM) as a mobile phase on a series of PSS Gram columns (Agilent Technologies, Mainz, Germany) with a flow rate of 1.0 mL min^−1^. Measured data were analyzed by ASTRA 8.1 (Wyatt) software and LabSolutions 5.106 (Shimadzu). The refractive index increment *dn*/*dc* was set at approximately 0.1 mL g^−1^.

### 2.8. UV-VIS Spectrophotometry

UV/VIS spectrophotometry (JENWAY 7415 nano, P-Lab, Prague, Czech Republic) was used for the determination of the molar content of -NHNH_2_ and -NH_2_ groups alongside the polymer chain of **P1** and **P2** according to the 2,4,6-trinitrobenzene-1-sulfonic acid (TNBSA) assay method, described elsewhere [30]. The amount of bound porphyrin (wt. %) was determined in a MeOH/DMA (8/2) (*v*/*v*) mixture for **C1** and **C2**, and DMSO for **C3**, by using the molar absorption coefficients at λ = 416 nm as follows: 393,900 M^−1^ cm^−1^ for **D1**, 426,300 M^−1^ cm^−1^ for **D2**, and 311,700 M^−1^ cm^−1^ for TPPc.

### 2.9. NMR

For the characterization of TPP derivatives, ^1^H NMR spectra were recorded using a Bruker Avance Neo 400 spectrometer (Mannheim, Germany). The spectrometer frequency was set at 400 MHz or 600 MHz, with a 90° pulse width of 16.5 μs, a relaxation delay of 10 s, and an acquisition time of 3.28 s. The number of scans was set to 32. Samples (5.0 mg) were dissolved in the appropriate deuterated solvent (550 μL of DMSO-*d6*). Spectral evaluation was performed using TopSpin 4.1.3 software. **D1**: ^1^H NMR (600 MHz, 295 K, DMSO-*d6*): δ = 9.02–7.65 (m, 27 H, Ar), 4.43 (t, 2 H, *J *= 6.3 Hz, -OCH_2_CH_2_-), 2.75 (t, 2 H, *J* = 7.0 Hz, -CH_2_CH_2_C(O)-), 2.18 (s, 3 H, -CH_3_), 2.04 (p, 2 H, *J* = 6.7 Hz, -CH_2_CH_2_CH_2_-), and −2.93 (s, 2 H, -NH-) ppm. **D2**: ^1^H NMR (600 MHz, 295 K, DMSO-*d6*): δ = 9.03–7.63 (m, 31 H, Ar), 5.62 (s, 2 H, -CH_2_-), 2.62 (s, 3 H, -CH_3_), and −2.93 (s, 2 H, -NH-) ppm. **D3**: ^1^H NMR (400 MHz, 300 K, DMSO-*d6*): δ = 9.12–7.61 (m, 27 H, Ar), 4.70 (t, 2 H, *J* = 7.1 Hz, -CH_2_CH_2_-), 3.73 (t, 2 H, *J* = 7.1 Hz, -CH_2_CH_2_-), and −2.92 (s, 2 H, -NH-) ppm.

### 2.10. Release of dTPP from the pH-Degradable Hydrazone Conjugates (C1 and C2)

The release of dTPP was carried out by incubating **C1** and **C2** containing pH-degradable hydrazone bonds in 0.1 M PB solutions of pH 5.0 and 7.4 at 37 °C. DMSO 5% (*v*/*v*) was added for better dissolution. At each chosen timepoint, an aliquot of the sample was rigorously shaken with CHCl_3_ to extract the released dTPP. CHCl_3_ was then evaporated, and dTPP was redissolved in DMF and analyzed by a HPLC LC10 system, equipped with an SPD-M20A detector (Shimadzu, Japan) and COSMOSIL 5C4-AR-300 column (Nacalai Tesque, INC. Kyoto, Japan). Water/ACN 95/5 with 0.1% TFA and DMF/ACN 50/50 with 0.1% TFA were used as an eluent with a 50–100% gradient at a flow rate of 2.5 mL min^−1^.

Calibration curves using the relative area of peaks (absorption maximum at λ = 416 nm) corresponding to different concentrations of dTPPs were analyzed in triplicate, and used later for the calculation of the released amount of dTPPs. The release was expressed relatively to the total dTPP content in the particular conjugate. The theoretical maximal amount of dTPP, calculated based on the dTPP’s content and the calibration curve, agreed with released dTPP after 4 h incubation at pH 1.7.

### 2.11. Determination of Changes in Micellar Size

The effect of dTPP release from conjugates **C1**–**C3** on the hydrodynamic diameter of micelles was evaluated by incubating the conjugates at 37 °C with a concentration of 2.0 mg mL^−1^ in 0.1 M PB of pH 5.0. After 4 h, 24 h, and 48 h, the incubated samples were filtered with 0.22 μm PVDF filter and their *D*_H_ was measured by DLS. A fluorescence filter was used during the measurement.

### 2.12. Fluorescence Spectroscopy Measurements

The fluorescence emission spectra were recorded on a spectrofluorometer JASCO FP-6200 (Tokyo Japan) and processed with Spectra ManagerTM software. The conjugates were dissolved in PB of pH 7.4, containing 5% (*v*/*v*) of DMSO, and TPPc was measured in DMSO for comparison. To determine the effect of the dTPP release, conjugates were incubated at 37 °C for 24 h in PB of pH 5.0 (with 5% DMSO (*v*/*v*)), in the presence of 0.1% SDS (*w*/*v*). The solutions, prepared in concentrations equivalent to 5.0 μg mL^−1^ of TPPc, were excited at 514 nm, and the emission spectra within the 550–800 nm region were recorded. More in depth evaluation can be found in our previous study [23].

### 2.13. In Vitro Cytotoxicity

The cytotoxicity of free TPPc and conjugates **C1**–**C3** was evaluated on mouse colon cancer C26 cells. Cells were cultivated in RPMI-1640 with 10% fetal bovine serum in phosphate-buffered saline (PBS, Nichirei Biosciences INC., Tokyo, Japan) at 37 °C under 5% CO_2_. Measurement was conducted in 96-well plates (each group consisted of 8 wells) with 5000 cells per well. After 24 h of incubation, TPPc or conjugates **C1**–**C3** were added at different concentrations. After 24 h of treatment, the media were removed and cells were washed several times by PBS and poured over with fresh medium. Irradiation was carried out for 5 min with a blue light at 420 nm (TL-D; Philips, Eidhoven, The Netherlands) and 1.0 J cm^−2^. After another 24 h, the viability of cells was quantified by MTT assay [31].

### 2.14. Intracellular Uptake

An intracellular uptake study was conducted on mouse colon cancer C26 cells. A total of 50,000 cells were cultivated in 16-well plates (each group consisted of 4 wells). After 12 h of incubation, conjugates **C1**–**C3** in PBS or free TPPc dissolved in DMSO were added to the cells at a concentration equivalent to 25 µg mL^−1^ TPPc. At predetermined timepoints, the drug-containing medium was removed, and cells were washed twice with PBS. Cells were then lysed using 1 mL of lysis buffer (4 N HCl in 70% EtOH) and heated at 70 °C for 15 min. The lysate was centrifuged at 15,000 rpm for 3 min, and the supernatant was collected for fluorescence measurement. Fluorescence emission was recorded at 650 nm with excitation at 416 nm using a multi-mode plate reader (Infinite M200 Pro, TECAN, Kawasaki, Japan).

### 2.15. Electron Spin Resonance Spectroscopy (ESR) for Singlet Oxygen Production

The generation of singlet oxygen, O_2_(^1^Δ_g_), by conjugates **C1**–**C3** upon light irradiation was analyzed by evaluating the ESR spectra of solutions containing the conjugates along with TMP, a spin trap selective for O_2_(^1^Δ_g_). The data were collected on ESR spectrometer JES FA-100 (JEOL, Tokyo, Japan) at 25 °C. Sample solutions containing conjugates (equivalent to 40 μg mL^−1^ of TPPc) were dissolved in 100 mM sodium PB with a pH of 5.0, with the addition of 0.1% Tween-20 or 50% DMSO and 20 mM of TMP. The samples were placed in flat quartz cells (Labotec, Tokyo, Japan) and irradiated using xenon light source MAX-303 (90 mW cm^−2^, 400–700 nm) (Asahi Spectra, Tokyo, Japan) for 300 or 150 s. The ESR spectrometer was typically set to a microwave power of 1.0 mW, an amplitude of 100 kHz, and a field modulation width of 0.1 mT.

### 2.16. In Vivo Biodistribution and PDT of Polymer Conjugates

Six-week-old male ddY mice were obtained from SLC (Shisuoka, Japan). All animals were housed under controlled conditions at 22 ± 1 °C with 55 ± 5% relative humidity and a 12 h light/dark cycle. All experiments were approved by the animal ethics committees (approved on April 1, 2014, no. 2024-P-005) and conducted in accordance with the Laboratory Protocol for Animal Handling of Sojo University. The endpoint of the experiment was governed by the tumor volume (up to ~2000 mm^3^). The mice were sacrificed by euthanasia using isoflurane.

Mouse sarcoma S180 cells (2 × 10^6^), maintained through weekly passages in mouse ascites, were subcutaneously implanted into the dorsal skin of ddY mice to establish the S180 solid tumor model. For the body distribution study, when tumors reached a diameter of approximately 10–12 mm, the mice were randomly grouped, with each group consisting of 4 mice, which, based on our experience, was sufficient to obtain statistically significant data while minimizing animal use. Mice with tumor sizes smaller than 10 mm or larger than 12 mm were excluded from this study. To minimize potential confounders, all mouse cages were placed in the same position on the shelf, and the mice were numbered according to the order of injection.

The conjugates, dissolved in physiological saline, were injected via i.v. into the tail vein at a dose of 2.5 mg kg^−1^ (equivalent to TPPc). After 24 h, the hair of mice around the tumor was shaved and, under anesthesia with isoflurane gas, the mice were subjected to in vivo fluorescence imaging using an IVIS XR (Caliper Life Science, Hopkinton, MA, USA) with excitation of 640 nm and emission of 695–770 nm. Then, the mice were sacrificed, tumors and other normal tissues were collected and subjected to ex vivo imaging, and the fluorescence intensity of each tissue/organ was quantified.

For the in vivo PDT study, when tumors reached a diameter of approximately 6–8 mm, the mice were randomly grouped, with each group consisting of 4 mice, to which different conjugates of indicated concentrations (1.25 or 2.5 mg kg^−1^, equivalent to TPPc) were administered intravenously. Then, 24, 48 or 72 h after the injection, the tumors were exposed to a xenon light (400–700 nm, 90 mW cm^−2^, MAX-303; Asahi Spectra, Tokyo, Japan) for 5 min at 24, 48, and 72 h post-injection. In some experiments, an additional irradiation 1 week after the drug administration was performed. Mice receiving i.v. injection of physiological saline were used as control. Tumor growth was monitored every 2 to 3 days by measuring the tumor size with a caliper. Tumor volume (V) was calculated using the formula V = (W^2^ × L)/2, where L represents the longitudinal cross-section and W represents the transverse section.

### 2.17. Statistical Evaluation

All results were presented as mean ± SD. Student’s t-tests were conducted to evaluate differences between two groups. Analysis of variance was performed for significance testing. The difference was considered statistically significant when *p* < 0.05.

## 3. Results and Discussion

Polymer-based theranostic systems represent a multifunctional approach that combines cancer therapy with diagnostic imaging. In this study, we focused on deep evaluation of polymer-based nanocarriers designed to transport porphyrin-based PSs and to examine how linker chemistry influences their activation, release behavior, and therapeutic performance under tumor-relevant conditions. In particular, we compared pH-responsive hydrazone linkers (of an aliphatic and aromatic nature) with stable amide linkers to elucidate how the design influences self-assembly, release behavior and photodynamic performance. Together with our previous work, focusing primarily on the photophysical and physicochemical properties of these systems, the present study aims to extend this understanding toward their biological behavior. We seek to provide a better insight into the underlying mechanisms linking the polymer design, mode of photosensitizer attachment, and the resulting therapeutic performance.

### 3.1. Synthesis and Characterization of Polymer Precursors

Both linear precursors **P1** and **P2** were prepared by controlled RAFT polymerization, yielding polymers with narrow dispersity and molecular weights suitable for glomerular filtration [32] in high yields (>80%). The polymers contained reactive functional groups distributed along the backbone—hydrazide groups in **P1** and amine groups in **P2**—which were successfully deprotected, as confirmed by the TNBSA assay described earlier. As a result, a sufficient amount of functional groups to enable subsequent conjugation of TPP derivatives was assured. Based on DLS measurement, both precursors formed random coils in aqueous solutions with hydrodynamic diameters (*D*_H_) around 9.5 nm and 7.4 nm for **P1** and **P2,** respectively, thus supporting their potential for renal elimination after fulfilling the role of PS carrier.

### 3.2. Synthesis and Characterization of Polymer Conjugates

Using carbodiimide chemistry, three TPP derivatives were successfully prepared, as confirmed by NMR and MALDI. Derivatives containing a keto group (**D1** with an aliphatic spacer and **D2** with an aromatic spacer) were subsequently reacted with hydrazide functionalities of the precursor **P1**, leading to the formation of hydrolytically degradable hydrazone bonds. In contrast, **D3** was conjugated to amino groups in **P2**, resulting in the formation of stable amide bonds. Resulting conjugates **C1**–**C3** contained comparable amounts of bound PS (see Table 2).

According to the SEC analysis, the conjugation did not lead to any significant shift of molecular weight or dispersity, as stated previously [23]. However, DLS measurements revealed the formation of micelles in aqueous conditions due to the introduction of hydrophobic dTPP into the hydrophilic polymer, which consequently resulted in an increase of hydrodynamic diameter compared to the polymer precursors (Table 2).

In our previous study [23], we also demonstrated that the nature of the spacer between TPPc moieties and the polymer backbone influences not only the size of formed micelles, but their stability in aqueous conditions as well. The longer and more flexible the spacer (**C1** > **C2** > **C3**) inserted, the easier and stronger the micellar self-assembly is, expressed by a lower critical micellar concentration (Table 2). Clearly, the long aliphatic spacer enables the rotation of large TPPc molecules, facilitating their stacking and forming the sandwich-like core of the micelle. In contrast, the more rigid aromatic spacer or the short amide bond restrict molecular rotation, preventing more efficient assembly of TPPc molecules inside the micelle core. As a result, these systems exhibit significantly lower kinetic stability. Overall, these findings indicate that **C1** shows the strongest intermolecular organization of TPPc within the micellar core, followed by **C2**, while **C3** exhibits the weakest organization among the proposed systems.

### 3.3. Release of dTPPs from the pH-Responsive Polymer Conjugates

**Figure 3:**
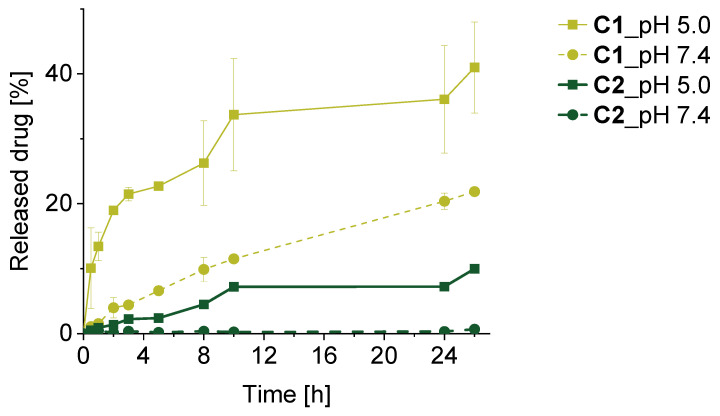
dTPP release from polymer conjugates **C1** and **C2** incubated in 0.1 M PB of pH 5.0 and pH 7.4 with 5% of DMSO (*v*/*v*) at 37 °C. Data are expressed as mean ± SD, *n* = 3.

The release of dTPPs was evaluated in PB with a pH of 5.0, mimicking the acidic lysosome environment inside tumor cells, and a pH of 7.4, modeling the neutral blood conditions, at 37 °C with 5% DMSO (*v*/*v*). A pH-dependent release profile of dTPPs from conjugates containing pH-labile hydrazone bonds was observed, with significantly faster release in pH 5.0 when compared to pH 7.4. The spacer’s structure, aliphatic (**C1**) vs. aromatic (**C2**), considerably influenced the hydrolytic stability of the hydrazone bond and the dTPP release (Figure 3). These results indicate that the local chemical environment of the hydrazone bond plays a key role in modulating its hydrolytic stability and, consequently, dTPP availability.

Although micelles become diluted upon intravenous administration, many polymeric micelles exhibit sufficient kinetic stability to persist in circulation. However, the extent of micelle disassembly in the bloodstream versus the tumor microenvironment remains uncertain, and the following mechanism should therefore be considered as a plausible model. Under physiological conditions, release is likely governed by limited water accessibility to the hydrazone bond within the hydrophobic micellar core, together with its intrinsic hydrolysis rate. Core hydration and polymer packing density thus play key roles in modulating the slow release of dTPP in circulation. In the tumor environment, increased core hydration and mildly acidic conditions may enhance the accessibility and cleavage of hydrazone bonds, promoting dTPP release. The released hydrophobic dTPP can subsequently interact with cellular membranes and be internalized. Overall, dTPP activation is expected to result from a combination of extracellular and intracellular processes.

### 3.4. Changes in Micellar Size upon dTPP Release

The effect of the dTPP release on the hydrodynamic diameter of micelles was assessed under simulated release conditions by dissolving conjugates **C1**–**C3** at a concentration of 2.0 mg mL^−1^ in 0.1 M PB (pH 5.0) and incubating the samples at 37 °C for 48 h. It was hypothesized that gradual dTPP release would lead to a corresponding decrease in micelle size. For conjugate **C1**, which contains the aliphatic spacer between the TPP moiety and the polymer backbone, micelle disintegration was observed after just 4 h of incubation. This was accompanied by the appearance of a smaller-size population, corresponding to the polymer carrier (see Figure S1). In contrast, **C2**, which exhibits a slower dTPP release, showed no distinct smaller-size population; only a minor shift to the left in the peak position was noted. As expected, the stable, non-releasing conjugate **C3** showed no observable changes in micellar size over the incubation period. These results confirm that the release of dTPP can trigger micelle disintegration, enabling the subsequent clearance of the polymer carrier from the body after it has fulfilled its role as a PS carrier.

### 3.5. Fluorescence Spectroscopy

**Figure 4:**
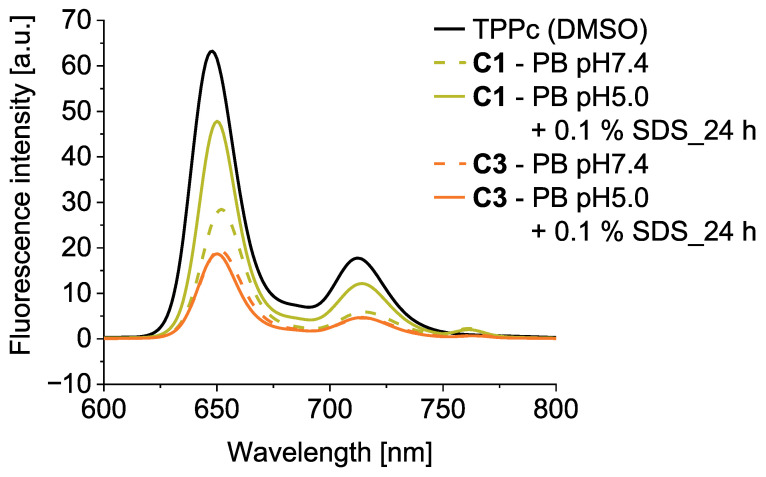
Fluorescence spectra of conjugates **C1** and **C3** in PB at a pH of 7.4 and after 24 h incubation in PB at a pH of 5.0 at 37 °C, compared to pure TPPc in DMSO. All the solutions were prepared at 0.005 mg mL^−1^ TPPc equivalent and excited at 514 nm. A total of 5% DMSO (*v*/*v*) was added for better dissolution.

The micelle formation in PB (pH 7.4) with 5% (*v*/*v*) DMSO resulted in the assembly of the porphyrin units within the hydrophobic micellar core and partial fluorescence quenching (Figure 4). These quenching effects were comparable for both **C1**, with cleavable hydrazone bonds, and **C3**, with stable amide bonds. Unlike for **C3**, the **C1** fluorescence intensity increased after incubation at 37 °C for 24 h in PB with a pH of 5.0 (with 5% (*v*/*v*) DMSO) and SDS to disrupt the micelles (Figure 4). This set up mimics the acidic environment of tumor tissues. The fluorescence increase for **C1** could be ascribed to the porphyrin release from this conjugate. Conjugate **C2** exhibited similar behavior to **C1**, showing a more pronounced increase in fluorescence compared to **C3** as a result of dTPP release (for clarity, these data are not included in the figure). We propose that both conjugates can be used for the successful irradiation of the tumor tissue. The fluorescence intensity of **C3** is not influenced by either the pH or micelle formation, making this conjugate a reliable probe for directly tracking the fate of the system.

### 3.6. In Vitro Cytotoxicity and PDT Effect of Polymer Conjugates

**Figure 5:**
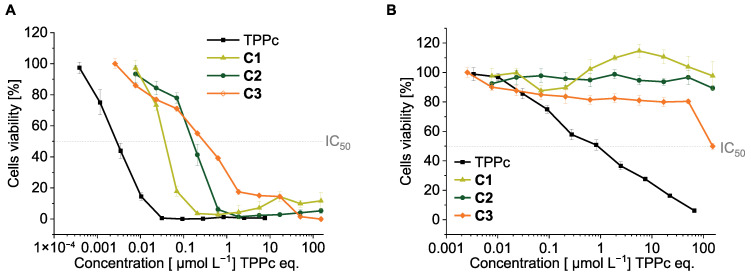
Cytotoxicity of TPPc and conjugates **C1**–**C3** towards C26 cells under irradiation at 420 nm (**A**) or compared with that in the dark (**B**). Values are mean ± SD (*n* = 8).

A previous study [23] demonstrated that conjugates **C1**–**C3** exhibited substantial in vitro PDT activity while showing markedly lower dark cytotoxicity compared to free TPPc. Among them, the pH-sensitive conjugate **C1** displayed the most promising in vitro PDT performance, characterized by a high phototoxicity index (PI). To further validate these findings, we repeated the in vitro cytotoxicity assays for the conjugates under both irradiation and dark conditions. The results are shown in Figure 5 and the IC_50_ as well as phototoxicity index are summarized in Table 3.

As shown in Figure 5A, all polymer conjugates, as well as TPPc, exhibited a dose-dependent PDT effect after irradiation. Compared to TPPc itself, the conjugates demonstrated a reduced PDT effect and associated cytotoxicity. This difference is likely due to the different cell-entering mechanisms of TPPc and its polymer conjugates. While free TPPc could easily enter the cells via the diffusion process, polymers must rely on a much slower process of endocytosis [33,34]. Notably, among the three conjugates, **C1** showed a stronger PDT effect than **C2**, with **C3** exhibiting the weakest cytotoxic response. This trend is in correspondence with the dTPP release experiments displayed in Figure 3. These in vitro findings support the assumption that the dTPP release rate plays a critical role in achieving a satisfactory PDT effect.

Importantly, free TPPc exhibited relatively high dark cytotoxicity, with an IC_50_ of approximately 1 µM. In contrast, all polymer conjugates showed markedly reduced dark toxicity, remaining essentially non-toxic up to ~100 µM (TPPc equivalent) for **C1**, **C2**, and **C3** (Figure 5B). Under irradiation, free TPPc displayed extremely potent phototoxicity, with an IC_50_ of approximately 0.003 µM, resulting in a PI of ~333. Notably, the polymer conjugates exhibited even higher PI values due to their substantially suppressed dark toxicity. In particular, **C1** showed a PI exceeding 2500, indicating a highly favorable therapeutic window. These results are consistent with our previous study [23] and further demonstrate that polymer conjugation effectively minimizes nonspecific dark toxicity while preserving strong light-triggered cytotoxicity. We hypothesize that they provide a safe drug delivery platform during circulation and distribution in the body, remaining non-cytotoxic until they accumulate in the tumor and are activated by light irradiation.

The cytotoxicity evaluation of stable amide conjugate post (**C3**) and prior (**C3WA**) blocking their amine groups by acetate-TT revealed an additional effect of residual amine groups. In **C3WA**, the IC_50_ values are shifted to slightly lower values compared to **C3**. The structures of **C3** and **C3WA**, as well as their dark/light cytotoxicity, are depicted in Figure S2.

### 3.7. Intracellular Uptake of Polymer Conjugates

**Figure 6:**
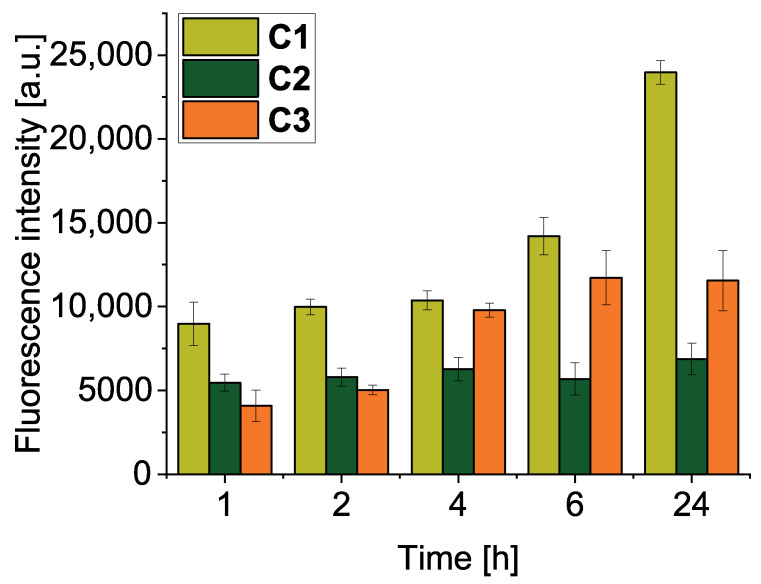
Intracellular uptake of conjugates **C1**–**C3** in C26 cells. Colon cancer C26 cells were incubated with different polymer conjugates for an indicated period of time. The amount of drug uptake by cells was determined by measuring the fluorescence intensity after the extraction from the cells, as described in Materials and Methods. Values are mean ± SD (*n* = 4).

We further investigated the intracellular uptake of TPPc and its conjugates by cultured C26 cells (Figure 6). Conjugate **C1**, which exhibits a higher dTPP release rate, showed faster and more extensive cellular internalization compared to **C2** and **C3**. This observation is consistent with the greater in vitro cytotoxicity of **C1** under irradiation (Figure 5A). We hypothesize that more extensive internalization of **C1** is caused by the combination of internalization of released **D1** and that bound on the polymer conjugate **C1**. Both **C2** and **C3** showed a steadier increase in intracellular uptake, with slightly higher levels observed for the hydrolytically stable **C3**. Notably, the internalization of porphyrin TPPc is approximately tenfold higher than that of polymer conjugates.

### 3.8. Generation of O_2_(^1^Δ_g_) by Conjugates upon Light Irradiation

**Figure 7:**
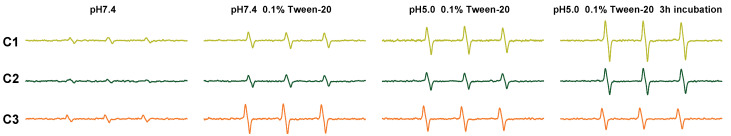
O_2_(^1^Δ_g_) generation by conjugates **C1**–**C3** as recorded by ESR spectroscopy. Polymer conjugates were dissolved in PBS with a pH of 7.4 or a pH of 5.0 in the absence/presence of 0.1% Tween-20. In some experiments, the samples were incubated for the indicated time at 37 °C. Light irradiation (90 mW cm^−2^) was carried out in the spectral region of 400–700 nm for 300 s. Photogenerated O_2_(^1^Δ_g_) was detected by TMP. Notably, ESR measurements of **C1** at a pH of 5.0 in the presence of Tween-20 (3 h incubation) were conducted with 50% DMSO to facilitate solubilization of the released dTPP.

In agreement with our previous findings [23], all conjugates (**C1**–**C3**) showed negligible O_2_(^1^Δ_g_) generation in aqueous solution due to micelle formation, indicating an “OFF state” caused by aggregation-induced quenching and limited oxygen accessibility (Figure 7 and Figure S3). Disruption of micelles by Tween-20 restored O_2_(^1^Δ_g_) production in all cases, confirming that micellar integrity is the primary determinant of photodynamic activity.

To simplify comparison, the key results from Figure S3 are summarized in Table 4, highlighting the effects of linker type, pH, and micelle disruption.

Among the conjugates, **C1** exhibited the highest O_2_(^1^Δ_g_) generation under acidic and micelle-disrupted conditions, consistent with its faster hydrazone cleavage, enhanced cellular uptake, and superior in vitro PDT efficacy (Figure 3, Figure 5 and Figure 6). **C2** showed similar but less pronounced behavior, while **C3** was insensitive to pH and relied solely on micelle disassembly for activation.

Notably, under certain conditions (i.e., at a pH of 5.0 with Tween-20, incubating for 3 h), **C1** showed reduced O_2_(^1^Δ_g_) generation despite rapid release (Figure S4). This effect is attributed to aggregation of the released hydrophobic photosensitizer (**D1**), which suppresses ROS production. Consistently, improved solubilization in 50% (*v*/*v*) DMSO restored O_2_(^1^Δ_g_) generation (Figure S4).

Overall, O_2_(^1^Δ_g_) generation in this system is governed by micelle stability, linker cleavage, and photosensitizer dispersion. The conjugates remain inactive during circulation and are activated upon micelle disruption and/or acidic conditions.

### 3.9. In Vivo Biodistribution of Polymer Conjugates

**Figure 8:**
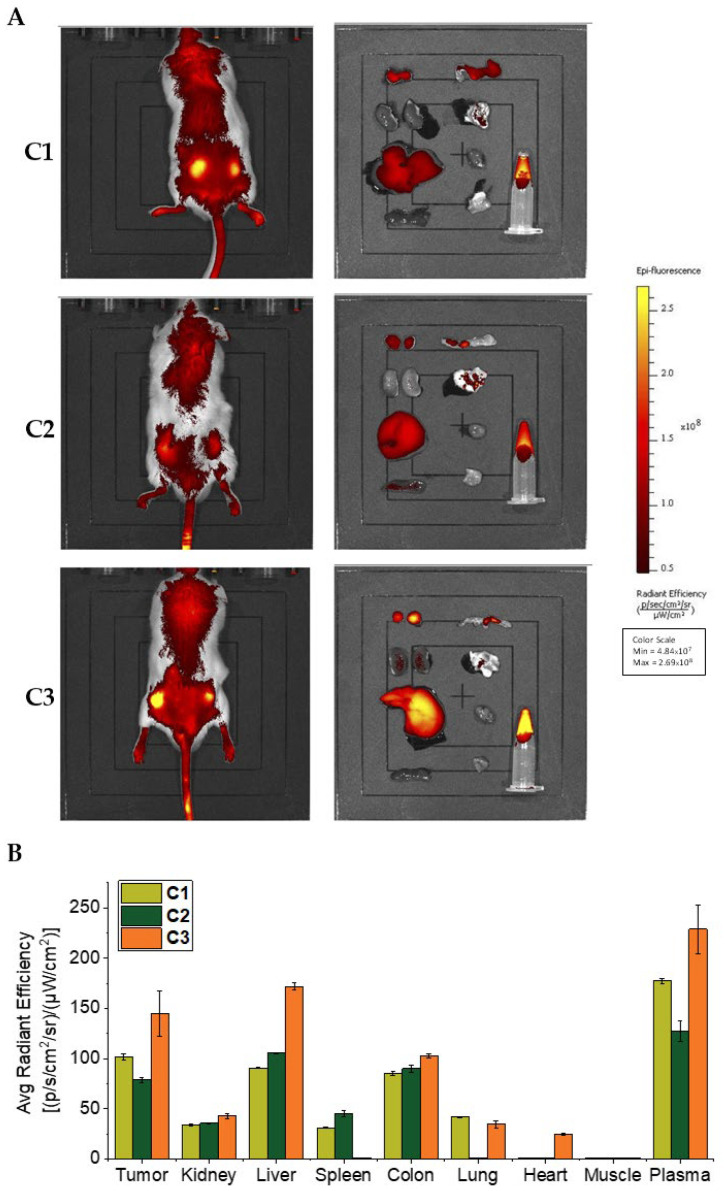
In vivo tumor imaging (**A**) and body distribution of polymer conjugates **C1**–**C3** (**B**) after i.v. injection in S180 tumor-bearing mice. ddY mice bearing mouse sarcoma S180 were used, with polymer conjugates (2.5 mg kg^−1^ TPPc equivalent) being i.v. injected when the tumor grew to size of about 10 mm in diameter. Twenty-four hours after i.v. injection, tumor imaging was carried out by an IVIS in vivo imaging system. Then, the mice were sacrificed and each tissue, including tumor tissue, was collected for fluorescence imaging by using the same system (**A**). The fluorescence intensity in each tissue was quantified to evaluate the tissue distribution of polymer conjugates (**B**). See text for details. Data are mean ± SD, *n* = 3–6.

One of the major advantages of polymeric drugs is the prolonged circulation time and tumor targeted accumulation compared to native drugs with a small molecular weight. Therefore, we measured the distribution of the polymer conjugates in the body by using in vivo imaging. As shown in Figure 8A, the application of **C1**–**C3** led to strong fluorescence of the tumor tissue 24 h after i.v. administration. The ex vivo imaging of each tissue revealed remarkable fluorescence intensities in the tumor and plasma, whereas very little fluorescence intensity was detected in most normal organs except for the liver, which is highly blood-prefaced and is the major organ to capture macromolecules (Figure 8A,B). When we compare the polymer conjugates, **C1** provides stronger fluorescence of the tumor and plasma than **C2**. This finding is consistent with the above-described release profiles (Figure 3) and O_2_(^1^Δ_g_) generation (Figure 7) studies, suggesting that tumor-responsive release may contribute to PDT performance. Notably, we observed that stable conjugate **C3** exhibited higher fluorescence intensity than **C1** and **C2**. Considering the low micellar stability of **C3**, it is likely present in the bloodstream as a random coil. We hypothesize that the enhanced accumulation of **C3** in tumors and the liver, as well as its prolonged plasma retention, may result from interactions between the porphyrin moieties of **C3** and hydrophobic domains on albumin. Such interactions could lead to the formation of larger conjugates, thereby promoting extended circulation and increased tumor accumulation. At the same time, the elevated liver signal indicates that this favorable tumor delivery is accompanied by less selective distribution.

### 3.10. In Vivo Antitumor PDT Effect of Polymer Conjugates

Based on the above results, we subsequently evaluated the in vivo PDT efficacy of polymer conjugates **C1**, **C2**, and **C3** in a mouse sarcoma S180 solid tumor model. This study was designed to systematically compare pH-responsive and non-responsive systems, as well as to distinguish between release-controlled and accumulation-driven therapeutic efficacy.

Our previous work employing PyFa as a PS, covalently bound via a degradable pH-sensitive hydrazone bond with an aliphatic spacer, showed the potential of a polymer-PyFa conjugate as a theranostic agent with dual imaging and therapeutic functions. A dosage as low as 2.5 mg kg^−1^ exhibited strong tumor-imaging capability and resulted in nearly complete photodynamic inhibition of tumors within 18 days post-treatment. Moreover, a reverse-dose dependency was observed, suggesting that effective PDT treatment relies on the balance between PS concentration and available molecular oxygen [8].

**Figure 9:**
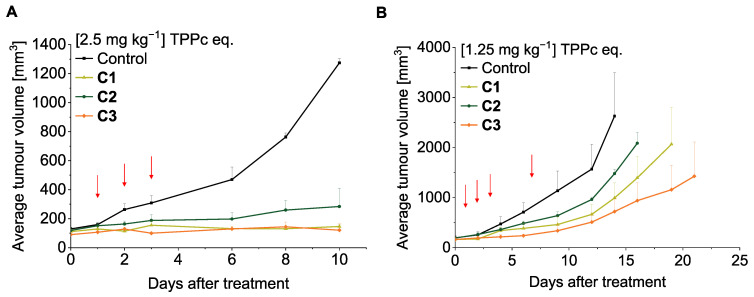
In vivo PDT effect of **C1**-**C3** conjugates upon irradiation by a xenon light source on a mouse sarcoma S180 solid tumor model. Indicated concentrations of 2.5 mg kg^−1^ (**A**) and 1.25 mg kg^−1^ (**B**) (TPPc equivalent) were injected via i.v. when tumor diameters reached 6∼8 mm. After 24 h, 48 h, and 72 h, light irradiation (90 mW cm^−2^, 5 min, 27 J cm^−2^) was performed. In some experiments, an additional irradiation was applied 1 week after the drug administration. The tumor growth was evaluated every 2 or 4 days. Data are mean ± SD; *n* = 8. See text for details.

Based on this study, a dosage of 2.5 mg kg^−1^ (TPPc equivalent) was selected as a starting point for in vivo evaluation. At this initial dosage, conjugate **C1** exhibited a slightly higher therapeutic effect than **C2**, likely due to its faster dTPP release. Notably, both **C1** and **C3** showed unexpectedly high treatment potency, with tumor growth being almost completely suppressed following the treatment (Figure 9A). To further investigate differences in therapeutic efficacy, the dosage was reduced to 1.25 mg kg^−1^ (TPPc equivalent) (Figure 9B). As anticipated from the in vitro results, conjugate **C1** produced a stronger PDT effect compared to **C2**. However, the overall in vivo trend differed from the in vitro behavior, as the stable amide conjugate **C3** yielded the most pronounced in vivo therapeutic effect. We attribute the superior treatment efficacy of **C3** to its enhanced PS delivery capability, leading to greater porphyrin accumulation in the tumor, as corroborated by the biodistribution data. At the same time, **C3** also showed stronger liver accumulation, indicating that improved efficacy was associated with a different distribution profile rather than with pH-responsive release alone. It is also noteworthy that the dose of 1.25 mg kg^−1^ (TPPc equivalent) was relatively low. Even under such conditions, all conjugates showed a significant therapeutic effect, indicating that both pH-responsive and non-cleavable systems can be active at relatively low doses, although through partially different dominant mechanisms. No signs of systemic toxicity of the treatment were observed, as evidenced by the stable body weight of treated mice (Figure S5).

Overall, the in vivo results indicate that the stable amide-linked conjugate **C3** achieved the strongest therapeutic effect under the tested conditions, likely due to its favorable tumor accumulation and sustained delivery. In contrast, the pH-responsive conjugates provided better control over release and activation-related behavior in vitro. Together, these findings show that therapeutic performance in this platform is determined by a balance between release properties, de-aggregation, and biodistribution, rather than by a single design parameter. The potential contribution of photothermal effects is an important consideration in PDT, particularly when infrared light is used. However, in this study, irradiation was performed using a light source in the 400–700 nm range, thereby excluding infrared wavelengths and minimizing possible photothermal contributions. We therefore attribute the observed therapeutic effects primarily to PDT-mediated mechanisms. Nevertheless, further studies are warranted to clarify this issue.

## 4. Conclusions

In this study, we report the deep evaluation of polymer-based nanomaterials as polymer-based nanomaterials for photodynamic therapy of sarcomas and related solid tumors. Tailored dTPP derivatives were covalently conjugated to highly biocompatible and water-soluble polymers via either pH-sensitive hydrazone bonds or stable amide linkages, resulting in self-assembled amphiphilic micellar structures with distinct biological behavior. All developed polymer constructs exhibited markedly reduced dark toxicity compared to free TPPc, confirming the advantage of polymer conjugation in minimizing non-specific toxicity during systemic delivery. At the same time, high phototoxicity indices were achieved, demonstrating efficient light-triggered activation. All systems accumulated in tumor tissue and showed significant antitumor activity even at low doses.

The hydrazone-linked conjugates showed clear pH-dependent behavior, including controlled release and enhanced singlet oxygen generation under acidic conditions, which was reflected in their favorable in vitro performance. However, in the in vivo tumor model, the amide-linked conjugate **C3** consistently achieved the highest therapeutic efficacy. This effect is attributed primarily to its improved tumor accumulation and prolonged retention, despite the absence of a cleavable linker. These findings indicate that, within this system, efficient delivery and biodistribution represent more critical determinants of therapeutic outcome than controlled release of the photosensitizer. At the same time, the increased liver accumulation observed for **C3** highlights an important limitation, suggesting that enhanced efficacy is accompanied by reduced distribution selectivity.

Taken together, this study shows that the design of polymer-based PDT systems cannot rely solely on stimulus-responsive activation strategies, but must instead consider the balance between stability, biodistribution, and photosensitizer availability. Under the conditions studied here, the non-cleavable amide-linked system proved to be the most effective, although further optimization of its distribution profile will be necessary. Further investigation of the underlying mechanisms, particularly the role of intracellular versus extracellular photosensitizer activity, may provide additional insight into how these systems can be rationally improved.

## Figures and Tables

**Table 1:** Physicochemical characterization of HPMA precursors.

Precursor	*M*_n_ (g mol^−1^) ^a^	*M*_w_ (g mol^−1^) ^a^	*Ð* ^a^	*D*_H_ ^a^ ± SD (nm)	Functional Group	Content of Func. Group (mol.%)
**P1**	34,600	36,400	1.05	9.5 ± 0.3	Hydrazide	4.5
**P2**	36,120	39,300	1.09	7.4 ± 0.1	Amine	4.0

^a^ *M*_n_ is the number-average molecular weight; *M*_w_ is the weight-average molecular weight; *Đ* is the dispersity; *D*_H_ is the hydrodynamic diameter measured by DLS.

**Table 2:** Physicochemical characterization of polymer conjugates ^a^.

Polymer Conjugate	Spacer	Content of dTPP (wt.%)	*D*_H_ ± SD (nm) ^a^	CMC (µg mL^−1^) ^b^
**C1**	5-hydroxy-2-pentanone	5.8 (**D1**)	16.4 ± 1.8	1.0
**C2**	1-(4-hydroxymethyl)phenyl)ethanone	5.8 (D2)	19.1 ± 0.5	4.5
**C3**	amide bond	6.2 (TPPc)	19.1 ± 2.0	>100

^a^ *D*_H_ is the hydrodynamic diameter measured by DLS in pH 7.4 /EtOH 5% (*v*/*v*) phosphate buffer with a concentration of 1.0 mg mL^−1^ at 25 °C. CMC was measured in 10 mM PBS by UV/VIS spectrophotometry. ^b^ CMC was measured in 10 mM PBS by UV/VIS spectrophotometry. More detailed CMC evaluation can be found in our previous paper [23].

**Table 3:** IC_50_ values and phototoxicity index for free TPP-COOH and conjugates **C1**–**C3**.

		TPP-COOH	C1	C2	C3
IC50 [μM] TPP-COOH eq.	Dark	1	>100	>100	>100
	Irradiation	0.003	0.04	0.18	0.30
Phototoxicity Index		333	>2500	>556	>333

**Table 4:** Summary of O_2_(^1^Δ_g_) generation behavior of conjugates (**C1**–**C3**).

Polymer Conjugate	Linker Type	Micelle Intact (No Tween-20)	Micelle Disrupted (Tween-20)	pH Dependence	Overall In Vitro PDT Activity
**C1**	Hydrazone (fast)	Negligible (OFF)	High (ON)	Strong	Highest
**C2**	Hydrazone (slow)	Negligible (OFF)	Moderate–high (ON)	Moderate	Moderate
**C3**	Non-cleavable	Negligible (OFF)	Moderate (ON)	None	Lower

## Data Availability

The datasets for this work are available from the authors upon request.

## Supplementary Materials

The following supporting information can be downloaded at: https://www.mdpi.com/article/10.3390/pharmaceutics18050531/s1: Figure S1, “Hydrodynamic diameter (*D*_H_) of **C1** (measured as number distribution) upon incubation at the concentration of 2.0 mg mL^−1^ in phosphate buffer of pH 5.0 with 5% (*v*/*v*) of DMSO for 0 h and 4 h.”; Figure S2, “Comparison of the cytotoxicity of **C3** and **C3WA** with protected and unprotected residual -NH_2_ groups, respectively, under irradiation at 420 nm (B) and in the dark (**C**). The protection of residual amine groups is depicted in (A).”; Figure S3, “ESR monitoring of O_2_(^1^Δ_g_) generation by **C1**–**C3** in the presence and absence of 0.1% Tween-20 at various pHs. The determination is based on the in situ reaction of O_2_(^1^Δ_g_) with a 2,2,6,6-tetramethyl-4-piperidone spin trap. The samples were irradiated using a xenon light source MAX-303 light source (90 mW cm^−2^, 400–700 nm) (Asahi Spectra, Japan) for 300 s.”; Figure S4, “ESR spectra of C1 at pH 5.0 under different conditions. Following incubation, the ESR signal decreased, which is considered to be due to aggregation of the released dTPP. In contrast, the addition of DMSO, which improves dTPP solubility, resulted in a marked increase in the ESR signal.”; Figure S5, “Body weight changes in mice after PDT treatment using **C1**–**C3**. Indicated concentrations of polymer conjugates (A, 2.5 mg kg^−1^; B, 1.25 mg kg^−1^ TPPc equivalent) were injected via i.v. when tumor diameters reached 6–8 mm. After 24, 48 h, and 72 h, light irradiation (90 mW cm^−2^, 5 min, 27 J cm^−2^) was performed. In some experiments, an additional irradiation was applied 1 week after the drug administration. The body weights of mice were evaluated every 2 or 4 days. Data are mean ± SD; *n* = 4–8. See text for details.”.

## Abbreviations

The following abbreviations are used in this manuscript:

ACN	acetonitrile
ACVA	4,4′-Azobis(4-cyanovaleric acid)
AIBN	2,2′-azobisisobutyronitrile
carboxyethyl-TTc-ACVA	4-(2-carboxyethylsulfanylcarbothioylsulfanyl)-4-cyanopentanoic acid
CMC	critical micellar concentration
CTA	chain transfer agent
*Đ*	dispersity
DAMPs	damage-associated molecular patterns
DCM	dichloromethane
*D*_H_	hydrodynamic diameter
DIPEA	*N*,*N*-diisopropylethylamine
DLS	dynamic light scattering
DMA	*N*,*N*-dimethylacetamide
DMAP	4-(dimethylamino)pyridine
DMF	*N*,*N*-dimethylformamide
DMSO	dimethyl sulfoxide
dTPP	derivative of TPP-COOH
EADS	evolution-associated difference spectra
EDC	*N*-(3-dimethylaminopropyl)-*N*′-ethylcarbodiimide hydrochloride
EPR	enhanced permeability and retention
ESR	electron spin resonance
EtOH	ethanol
HPLC	high-performance liquid chromatography
HPMA	*N*-(2-hydroxypropyl)methacrylamide
Ma-Ahx-NHNH-Boc)	*N*-(*tert*-butoxycarbonyl)-*N*′-(6-methacrylamidohexanoyl)hydrazine
Ma-AP-NH-Boc	*N*-(3-*tert*-butoxycarbonyl-aminopropyl)methacrylamide
MALDI	matrix-assisted light desorption/ionization
MeOH	methanol
*M*_n_	number-average molecular weight
*M*_w_	weight-average molecular weight
NMR	nuclear magnetic resonance
PB	phosphate buffer
PBS	phosphate buffer saline
PDT	photodynamic therapy
PS	photosensitizer
PyFa	pyropheophorbide-a
RAFT	radical reversible addition–fragmentation chain transfer
ROS	reactive oxygen species
SD	standard deviation
SDS	sodium dodecyl sulfate
SEC	size exclusion chromatography
*t*-BuOH	*tert*-butanol
TMP	2,2,6,6-tetramethyl-4-piperidone
TEMPO	4-oxo-2,2,6,6-tetramethylpiperidine-1-oxyl
TFA	trifluoroacetic acid
TNBSA	4,6-trinitrobenzene-1-sulfonic acid
TPP-COOH	5,10,15-triphenyl-20-(4-carboxyphenyl) porphyrin
TT	2-thiazoline-2-thiol
V-70	2,2’-azobis(4-methoxy-2,4-dimethylvaleronitrile)
