# Supplementary material for: Polymeric Theranostics with Tetraphenylporphyrin for Effective Low-Dose Photodynamic Cancer Therapy
Source: Pharmaceutics. 2026 Apr 27;18(5):531. doi: 10.3390/pharmaceutics18050531 (PMC13210605; doi:10.3390/pharmaceutics18050531)
Supplement: Supplementary file 1 [file pharmaceutics-18-00531-s001.zip › pharmaceutics-4206523-supplementary.pdf]

pharmaceutics

Supporting information

Polymeric Theranostics with Tetraphenylporphyrin for Effective Low-Dose Photodynamic Cancer Therapy

Alžběta Turnovská [1,†], Shanghui Gao [2,†], Marina Rodrigues Tavares [1], Jan Hynek [3], Kamil Lang [3], Jun Fang [2,4,*] and Tomáš Etrych [1,*]

[1] Institute of Macromolecular Chemistry, Czech Academy of Sciences, 16200 Prague, Czech Republic; turnovska@imc.cas.cz (A.T.); tavares@imc.cas.cz (M.R.T.)
[2] Laboratory of Microbiology and Oncology, Faculty of Pharmaceutical Sciences, Sojo University, Kumamoto 860-0082, Japan; gaoshanghui94@gmail.com
[3] Institute of Inorganic Chemistry, Czech Academy of Sciences, 250 68 Husinec-Řež, Czech Republic; hynek@iic.cas.cz (J.H.); lang@iic.cas.cz (K.L.)
[4] Department of Toxicology, School of Public Health, Anhui Medical University, Hefei 230032, China
[*] Correspondence: fangjun@ph.sojo-u.ac.jp (J.F.); etrych@imc.cas.cz (T.E.)
[†] These authors contributed equally to this work.

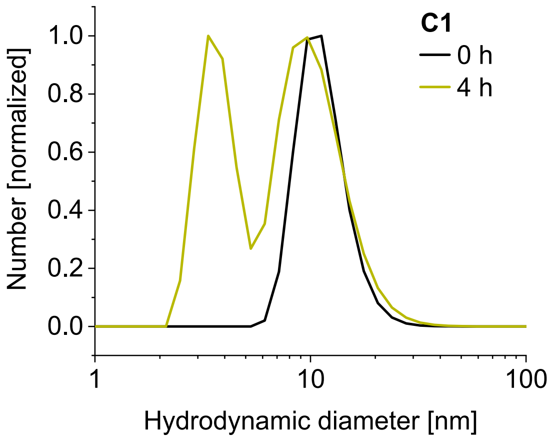

Figure S1. Hydrodynamic diameter (D_H) of **C1** (measured as number distribution) upon incubation at the concentration of 2.0 mg mL^{-1} in phosphate buffer of pH 5.0 with 5 % (*v*/*v*) of DMSO for 0 h and 4 h.

 https://doi.org/10.3390/pharmaceutics18050531

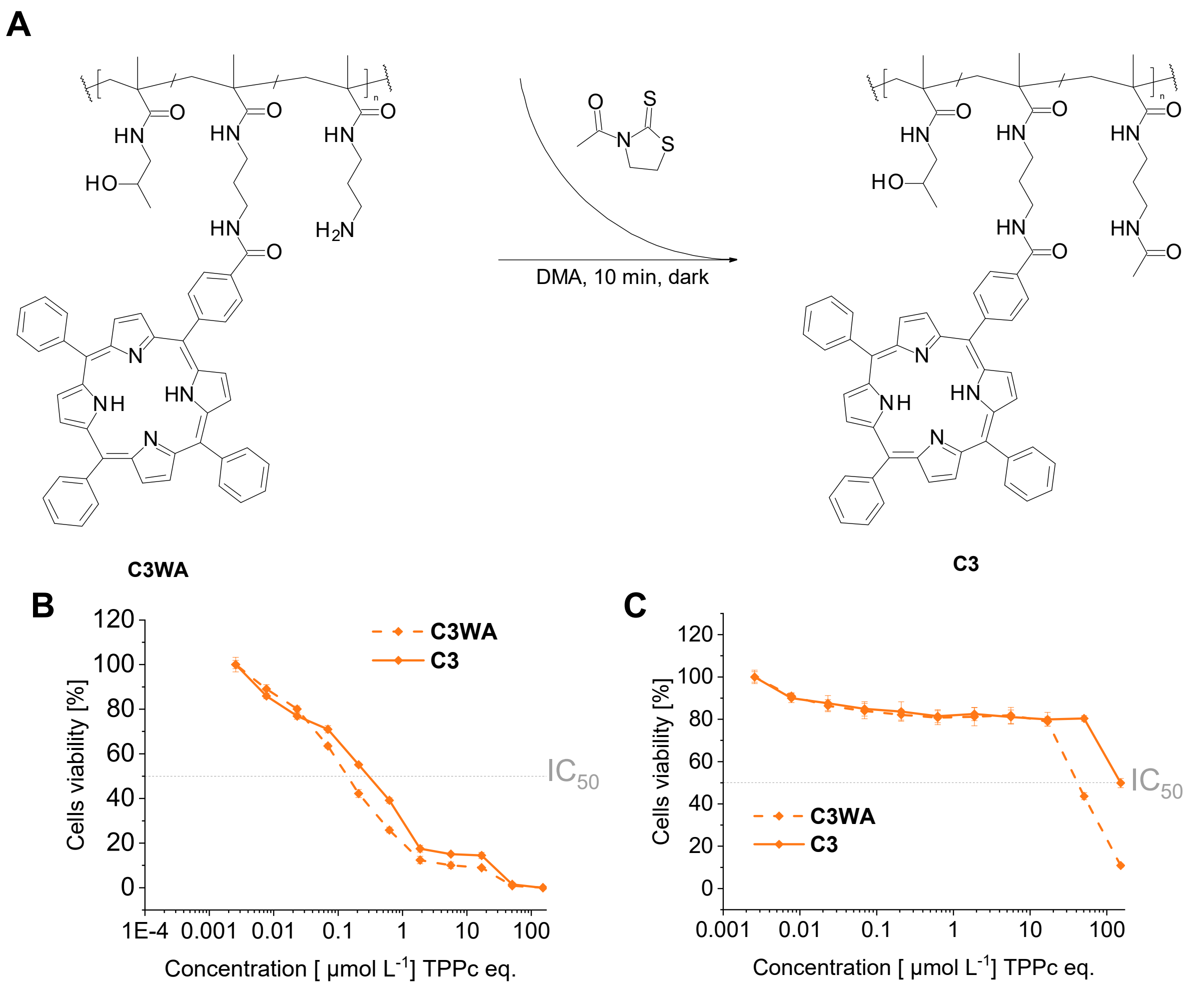

Figure S2. Comparison of the cytotoxicity of **C3** and **C3WA** with protected and unprotected residual -NH_2 groups, respectively, under irradiation at 420 nm (**B**) and in the dark (**C**). The protection of residual amine groups is depicted in (**A**).

https://doi.org/10.3390/pharmaceutics18050531

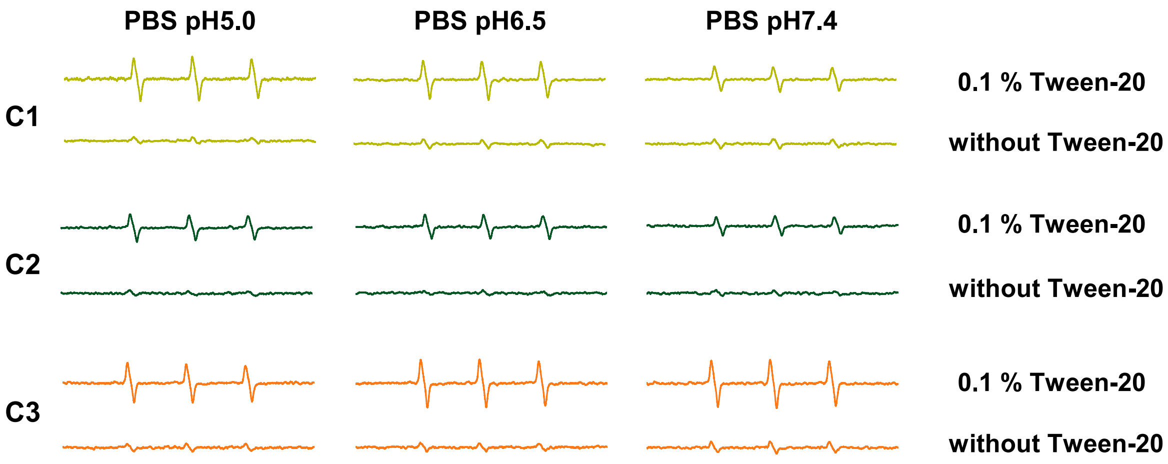

Figure S3. ESR monitoring of $O_2(^1\Delta_g)$ generation by **C1**-**C3** in the presence and absence of 0.1 % Tween-20 at various pHs. The determination is based on the in-situ reaction of $O_2(^1\Delta_g)$ with a 2,2,6,6-tetramethyl-4-piperidone spin trap. The samples were irradiated using a xenon light source MAX-303 light source (90 mW cm^{-2}, 400 - 700 nm) (Asahi Spectra, Japan) for 300 s.

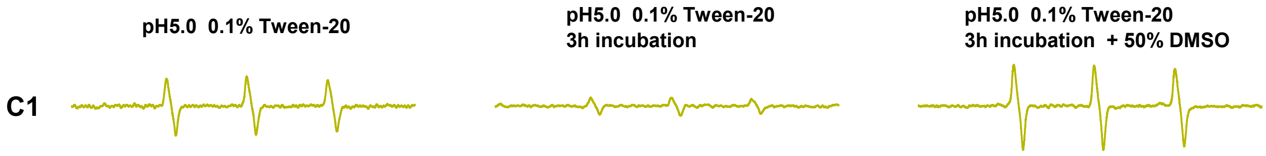

Figure S4 ESR spectra of **C1** at pH 5.0 under different conditions. Following incubation, the ESR signal decreased, which is considered to be due to aggregation of the released dTPP. In contrast, the addition of DMSO, which improves dTPP solubility, resulted in a marked increase in the ESR signal.

https://doi.org/10.3390/pharmaceutics18050531

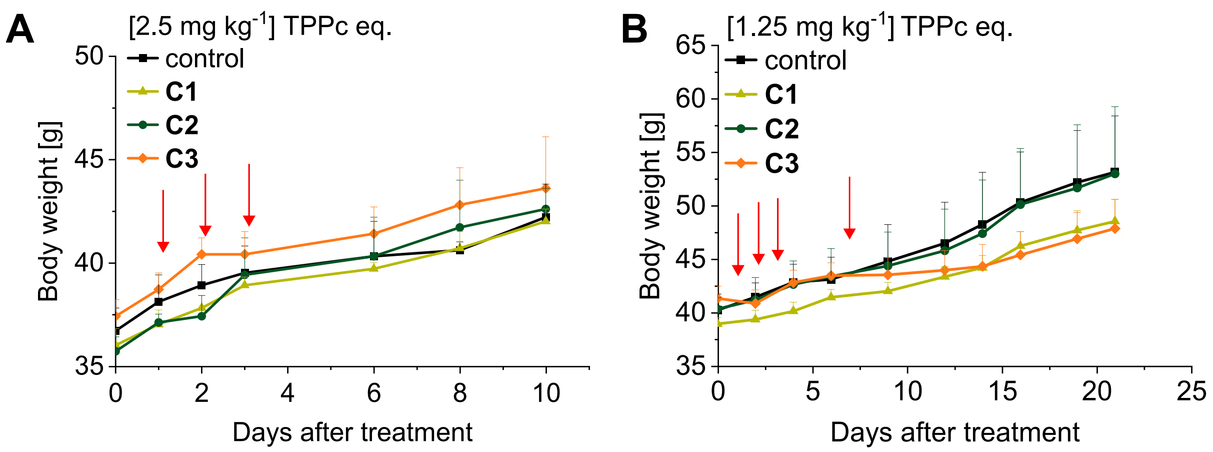

Figure S5. Body weight changes of mice after PDT treatment using **C1** – **C3**. Indicated concentrations of polymer conjugates (**A**, 2.5 mg kg^{-1}; **B**, 1.25 mg kg^{-1} TPPc equivalent) were injected i.v. when tumor diameters reached 6-8 mm. After 24, 48 h, and 72 h, light irradiation (90 mW cm^{-2}, 5 min, 27 J cm^{-2}) was performed. In some experiments, an additional irradiation was applied 1 week after the drug administration. The body weights of mice were evaluated every 2 or 4 days. Data are means ± SD; n=4–8. See text for details.

Disclaimer/Publisher's Note: The statements, opinions and data contained in all publications are solely those of the individual author(s) and contributor(s) and not of MDPI and/or the editor(s). MDPI and/or the editor(s) disclaim responsibility for any injury to people or property resulting from any ideas, methods, instructions or products referred to in the content.

https://doi.org/10.3390/pharmaceutics18050531
